# Genome-environment association analysis reveals climate-driven adaptation of chickens

**DOI:** 10.1186/s12711-025-00989-9

**Published:** 2025-07-22

**Authors:** Xiurong Zhao, Jinxin Zhang, Junhui Wen, Xinye Zhang, Haiying Li, Huie Wang, Tao Zhu, Changsheng Nie, Xinghua Li, Weifang Yang, Guomin Cao, Wenjie Xiong, Xue Wang, Zhonghua Ning, Lujiang Qu

**Affiliations:** 1 College of Grassland Science and Technology, China Agricultural University, Beijing, 100193 China; 2 Fisheries Science Institute, Beijing Academy of Agriculture and Forestry Sciences, Beijing, 100097 China; 3 Institute of Animal Husbandry and Veterinary Medicine, Beijing Academy of Agricultural and Forestry Sciences, Beijing, 100097 China; 4 National Engineering Laboratory for Animal Breeding, College of Animal Science and Technology, China Agricultural University, Beijing, 100193 China; 5 College of Animal Science, Xinjiang Agricultural University, Urumqi, 830000 China; 6 Key Laboratory of Protection and Utilization of Biological Resources in Tarim Basin, Xinjiang Production and Construction Corps, Tarim University, Alar, 843300 China; 7 Beijing Municipal General Station of Animal Science, Beijing, 100107 China; 8 Animal Husbandry Station of Fangchenggang, Guangxi, 538001 China; 9 Animal Disease Prevention and Control Center of Fangchenggang, Guangxi, 538001 China; 10 VVBK Animal Medical Diagnostic Technology, Beijing, 102600 China

## Abstract

**Background:**

Domestic chickens are one of the most widely raised and distributed bird species, exhibiting remarkable environmental adaptability, which makes them valuable model organisms for investigating the genetic mechanisms underlying climate adaptation. This study aimed to enhance our understanding of adaptive mechanisms in chickens by jointly analyzing genomic variations and climatic variables related to temperature and precipitation. To this end, whole-genome sequencing data were collected from 199 indigenous domestic chickens raised under diverse environmental conditions worldwide, and three genome-environment association analyses were performed.

**Results:**

We identified 184 genes potentially associated with climate adaptation in chickens. Among these, the *TSHR* gene may play multiple roles in adaptation driven by different climatic factors. Immune-related genes also appear to contribute to climate adaptation in chickens. By calculating the allele frequencies of single nucleotide polymorphisms (SNPs) within candidate genes associated with temperature and precipitation adaptation, we identified five SNPs within four genes (*ZNF536*, *ENSGALG00000049158*, *PAPPA*, and *EHMT1*) that exhibited distinct geographic distribution patterns. Extended haplotype homozygosity (EHH) analysis of these SNPs revealed that haplotypes carrying the mutant allele exhibited slower decay in EHH compared to those carrying the wild-type allele. These results further indicate that the loci have experienced strong selective pressures, suggesting that the associated genes may play crucial roles in climate adaptation in chickens.

**Conclusions:**

Overall, this study provides new insights into the genetic mechanisms underlying climate adaptation in domestic chickens.

**Supplementary Information:**

The online version contains supplementary material available at 10.1186/s12711-025-00989-9.

## Background

Environmental pressures caused by multiple factors, including temperature and precipitation, are crucial drivers shaping the phenotypic and genetic diversity of organisms [1–4]. Such pressures can influence the reproduction and immune performance of animals, leading to decreased animal welfare and economic losses [5–7]. Therefore, identifying genetic variations driven by different environmental and climatic factors has crucial implications for understanding the mechanisms underlying species adaptation, and the potential to reveal key functional variants [8, 9].

Domestic chickens exhibit remarkable climate tolerance, making them valuable model organisms for studying climate-mediated adaptation. Chickens were domesticated from red junglefowl in Southeast Asia approximately 8000 years ago, a process closely tied to the development of human culture [10, 11]. As humans migrated and engaged in frequent terrestrial and maritime trade, chickens were gradually introduced to regions worldwide [12] and became one of the most widely distributed and extensively raised livestock species [13]. As of 2015, the Food and Agriculture Organization [14] reported that there were 1514 unique local chicken breeds worldwide. Chickens inhabiting different environments have developed distinct climate adaptation mechanisms.

Many studies have investigated the genetic basis of climate adaptation in chickens [12, 15–18]. Most of these employed simple and direct methods for grouping and comparing samples, such as contrasting populations from arid versus humid regions or high versus low altitudes and latitudes. Approaches based on population genetics [e.g., fixation index (FST), cross-population extended haplotype homozygosity (XP-EHH) and, haplotype-based Lewontin and Krakauer test (hapFLK)] have been widely used in these studies to detect genetic variations associated with climate adaptation [19, 20]. However, these methods do not account for environmental heterogeneity, potentially leading to a high rate of false positives [20]. Additionally, they cannot establish direct associations between the identified loci and specific environmental variables [4].

With the advancement of global climate monitoring technologies and data management, several mature climate databases have been established, including the National Ecosystem Science Data Center (http://www.nesdc.org.cn), Resource and Environment Science Data Center of the Chinese Academy of Sciences (https://www.resdc.cn), and WorldClim (https://worldclim.org) [21]. These databases compile and record detailed climate data characterized by a long temporal span, broad geographic coverage, high resolution, and diverse climatic variables (e.g., variables related to temperature, precipitation, and elevation). Such resources provide a vital foundation for in-depth explorations of the genetic mechanisms underlying climate adaptation in animals. Furthermore, the rapid development of high-throughput sequencing technologies has enabled researchers to uncover the genetic bases of climate adaptation in animals more efficiently and accurately.

In this context, landscape genomics has emerged as a new approach to exploring the molecular mechanisms of ecological adaptation [22, 23]. This approach facilitates the identification of genetic variations associated with particular climatic variables by integrating genetic data with climatic variables for genome-environment association (GEA) analysis [23]. Landscape genomics has been employed to study climate adaptation in plants (e.g., forest trees, rice, and *Circaeaster*), sheep, and goats [4, 24–27]. In poultry, Gheyas et al. investigated climate adaptation in chickens from Ethiopia and identified important environmental variables associated with habitat suitability [28]. However, given the diverse effects of different environmental pressures on climate adaptation in domestic chickens, integrating genetic and climatic data from chickens across different geographic regions is essential for deeper understanding of the mechanisms underlying their adaptability to various climates.

The climatic characteristics of various regions show significant differences, particularly in temperature, precipitation, and elevation. Among these factors, the distinct combinations of temperature and precipitation patterns have shaped a gradient of climatic conditions, ranging from hot-humid and hot-arid to cold-humid and cold-arid [28]. These diverse climatic gradients pose extensive environmental adaptation challenges for livestock production. Therefore, in the present study, we primarily investigated the mechanisms by which chickens adapt to temperature and precipitation. To this end, whole-genome sequencing data were collected from 199 indigenous domestic chickens raised under diverse environmental conditions in Asia, Africa, and North America. Subsequently, 19 climatic variables related to temperature and precipitation were integrated to perform GEA analysis, providing new insights into the genetic mechanisms underlying chicken adaptation.

## Methods

### Sampling and genome sequencing

In total, 199 indigenous domestic chickens with clear sampling locations and geographic origins were included in this study. They originated from East Asia, South Asia, Central Asia, Southeast Asia, Africa, and North America. Some of them were newly collected for this study, while others were obtained from previous studies [10, 18, 29–32]. Sample details are provided in Additional file 1 Table S1. For the newly collected samples, genomic DNA was extracted using the standard phenol–chloroform method, and whole-genome resequencing was performed on the Illumina HiSeq 2500 sequencing platform.

### Genomic variant calling

Firstly, quality filtering was conducted using fastp [33] to remove low-quality reads and those aligned to the barcode adapter. After quality filtering, the cleaned reads were aligned to the reference genome (Gallus gallus 6.0) using Burrows-Wheeler Aligner v. 0.7.17 [34]. Then, bam files were sorted using Samtools (v. 1.11) [35] and duplicate reads were marked using the “MarkDuplicates” function in Picard tools (v. 2.23.3) (https://broadinstitute.github.io/picard). Subsequently, the “BaseRecalibrator” and “ApplyBQSR” parameters of Genome Analysis Toolkit (GATK) v. 4.2 [36] were used to perform base quality recalibration of the reads. For genetic variant calling, the “Haplotypecaller” and “GenotypeGVCFs” functions of GATK were used, with default parameters. Finally, the “VariantFiltration” module of GATK was used to remove SNPs with “QUAL < 30.0 || QualByDepth < 5.0 || FisherStrand > 60.0 || RMSMappingQuality < 40.0 || MappingQualityRankSumTest < − 12.5 || ReadPosRankSumTest < − 8.0”. And SNPs with minor allele frequency (maf) less than 0.05 were further filtered using VCFtools v. 0.1.15 [37] with the parameter “–maf 0.05”.

### Genetic diversity and population structure analysis

To assess genetic diversity in chicken populations, we utilized various parameters, including observed heterozygosity (Ho) and expected heterozygosity (He), which were calculated using PLINK v. 1.9 [38]. Additionally, genome-wide nucleotide diversity (π) was determined using VCFtools. To avoid potential bias due to sequencing depth and population size [39–44], we estimated Ho, He, and π across 15 populations: XJBC, XJHT, BJ, HLJ, Hubei, Jiangsu, GX, Hainan, GD, YN, Sri_Lanka, Canada, AF_Horro, AF_Jarso, and Vietnam2.

We then carried out a population structure analysis. To begin, we used PLINK to perform linkage disequilibrium (LD) pruning with the parameters “–indep-pairwise 50 10 0.1” to reduce the influence of LD on the population structure. Following that, we conducted principal component analysis (PCA) with the same software. Additionally, we used Admixture software [45] with default parameters to investigate the ancestry of each individual, with the genetic clusters number of K ranging from 2 to 15.

### Environmental data and GEA analysis

Nineteen climatic variables with a spatial resolution of 30 arcseconds (~ 1 km^2^) were downloaded from the WorldClim database [21]. Of these, 11 are related to temperature and eight are related to precipitation. The climate data covered a 30-year span, from 1970 to 2000. For each sample in this study, we used the ArcGIS platform to extract the corresponding climatic variables based on the coordinates of the sampling locations. Further details on the climatic variables are provided in Additional file 1 Table S2.

Then, we used the LD-pruned SNP set to conduct three GEA analyses, including univariate latent-factor linear mixed model (LFMM), individual-based spatial analysis, and redundancy analysis (RDA), to identify climate-driven variants across the whole genome. 

LFMM can be used to identify candidate genetic variants that are strongly associated with environmental variables while correcting for background levels of population structure. In this method, genotypic data are treated as response variables, while environmental variables are considered fixed effects, while modeling population structure using latent factors [46]. In this study, we conducted LFMM analysis using the R package ‘LEA’ [47] to assess the relationship between genetic variants and 19 climatic variables. To account for population structure in the genotypic data, we performed LFMM with five latent factors, based on the number of ancestry clusters inferred by Admixture. For each climatic variable, five Markov Chain Monte Carlo (MCMC) runs with a burn-in of 5000 followed by 10,000 iterations were employed. Subsequently, we averaged the *P-*values from all five runs and adjusted them for multiple runs using a false discovery rate (FDR) correction of 5% as the significance level [24].

Individual-based spatial analysis was conducted to investigate the association between specific genetic loci and 19 climatic variables using a logistic regression model in Samβada [48], primarily with the univariate analysis module. In the univariate case, each model involving a genotype and an environmental variable is contrasted with a constant model, which assumes that the likelihood of the presence of the genotype is the same across all landscape locations, matching its frequency in the dataset. Significance was of what? Was assessed using both log-likelihood ratio (G) and Wald tests. Based on the principle of Bonferroni correction for multiple comparisons, we set the nominal significance threshold (α) to 0.01 and then obtained the significance threshold α’ as α/m, where m is the number of models that were fitted, i.e. the number of SNPs analyzed. The models with *P*-values (computed from G and Wald scores) ≤ α’ were considered significant [48] and the SNPs implicated in these models were inferred to be candidate loci related to climate adaptation.

RDA is a constrained ordination that models linear combinations of explanatory variables (e.g., environmental variables) and response variables (e.g., genetic variants), thereby identifying genetic variants associated with multiple environmental variables [49]. The use of highly correlated environmental variables may affect RDA results due to multicollinearity among the factors [50]. To avoid issues arising from multicollinearity, we first calculated the correlations among the 19 climatic variables and evaluated their ranked importance via gradient forest analysis using the R package ‘GradientForest’ [51]. After considering the correlations among the environmental factors and their ranked importance (see Additional file 2 Figure S1), we finally selected the following six variables with pairwise correlation coefficients |r|< 0.7 to perform RDA using the R package ‘vegan’ [52]: maximum temperature of warmest month (BIO5), mean temperature of wettest quarter (BIO8), mean temperature of coldest quarter (BIO11), precipitation of driest month (BIO14), precipitation seasonality (BIO15), and precipitation of warmest quarter (BIO18). Of these six variables, three are related to temperature and the other three are related to precipitation. In the RDA model, genetic variants were treated as response variables, while the six climatic variables served as explanatory variables. Latitude, longitude, and population structure were included as conditioning variables, the effects of which were ultimately removed from the analysis [52]. Significant SNPs associated with climatic variables were considered as those having loadings in the tails of the distribution, using a 3-standard deviation cut-off along one or more RDA axes.

### Candidate SNP annotation and candidate gene analysis

We annotated the significant SNPs identified by the three methods using the Biomart module of Ensembl (http://www.ensembl.org/biomart/martview). Subsequently, Kyoto Encyclopedia of Genes and Genomes (KEGG) analysis was conducted to investigate biological functions related to climate adaptation using KOBAS [53]. For genes detected by all three methods, we examined their overlap with quantitative trait loci (QTLs) previously reported to be related to chicken adaptation, including those recorded in the Chicken QTL database (https://www.animalgenome.org/cgi-bin/QTLdb/GG/index) and those identified in previous studies [12, 15, 16, 18, 28, 31, 54–63]. Using VCFtools, we analyzed the allele frequency distribution patterns of SNPs in candidate genes related to temperature and precipitation adaptation across 20 chicken populations: XJBC, XJBHT, BJ, HLJ, Hubei, Jiangsu, GX, Hainan, GD, YN, TW, Sri_Lanka, Canada, AF_Horro, AF_Jarso, Indonesia1, Indonesia2, Indonesia3, Vietnam1, and Vietnam2. Only populations with a minimum of five individuals were included in this allele frequency analysis. Additionally, we utilized the whole-genome sequencing data from 199 indigenous domestic chickens representing 35 populations involved in this study to evaluate the EHH patterns for haplotypes carrying the ancestral and derived alleles at SNPs using the R package ‘rehh’ [64]. Ancestral alleles are the reference alleles, while derived alleles are those that had undergone mutations compared to the reference genome.

## Results

### Genome sequencing and population structure

**Fig. 1:**
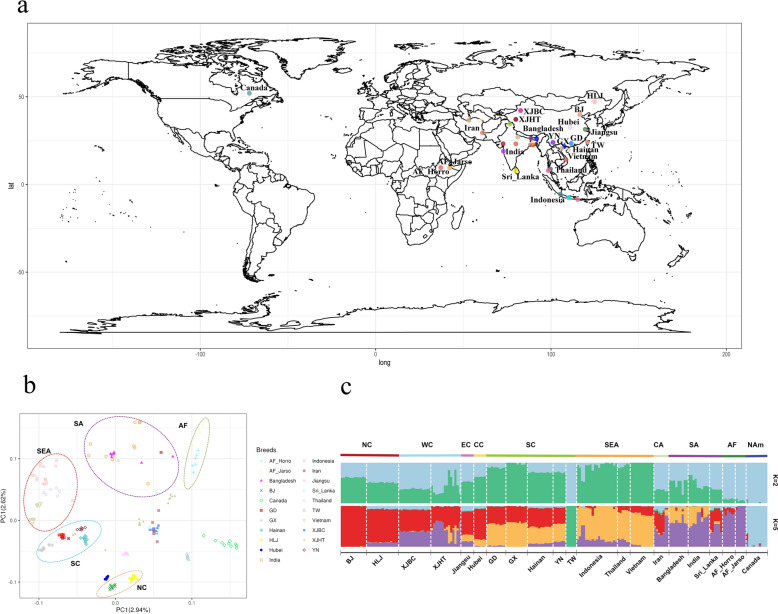
Population structure analysis of the evaluated indigenous chickens. **a** Sampling location and geographic origins of samples used for this study. A total of 199 chickens including 56 form Northern China (Baicheng Xingjiang, Hetian Xinjiang, Beijing, and Heilongjiang), 6 from Central China (Hubei), 6 from Eastern China (Jiangsu), 42 from Southern China (Guangxi, Hainan, Guangdong, Yunnan, and Taiwan), 25 from South Asia (Sri_Lanka, Bangladesh, and India), 7 from Central Asia (Iran), 10 from Canada (Canada), 11 from Africa (Horro, and Jarso), and 36 from Southeast Asia (Indonesia, Thailand, and Vietnam). Details on sample names and source are provided in Additional file 1 Table S1; **b** PCA plot of all samples. PC1, principal components one; PC2, principal components two. Chicken population from SEA, SC, NC, SA, AF were indicated by different circles. The abbreviations of samples and chicken population are provided in Additional file 1 Table S1. **c** Admixture result for K = 2 and K = 5. The abbreviations of samples and chicken population are provided in Additional file 1 Table S1

We analyzed the whole genome sequencing data from 199 indigenous domestic chickens across various environmental conditions (Fig. 1a). After aligning the reads to the reference genome, we obtained 20.5 billion mapped reads, with an average sequencing depth of 14.7× and an average mapping rate of 99.3% per individual (see Additional file 1 Table S3). After calling variants, we obtained 31.48 million SNPs. Following GATK hard-filtering, the number of SNPs was reduced to 29.54 million. After further filtering using the parameter “–maf 0.05” in VCFtools, we obtained 13.64 million SNPs. Susequently, we kept 12.84 million SNPs from the autosomes for analysis using VCFtools. The average SNP density per chromosome was 13,360 SNPs/Mb, with the lowest density observed on chromosome 22 (6868 SNPs/Mb) and the highest density on chromosome 6 (15,273 SNPs/Mb) (see Additional file 1 Table S4).

The results of the genetic diversity analysis for each chicken population are presented in Additional file 1 Table S5. We observed that the local chicken population in Sri Lanka had the highest observed heterozygosity (Ho) value at 0.295. In contrast, the Hainan local chicken population showed the highest expected heterozygosity (He) and nucleotide diversity (π), with values of 0.282 and 0.296, respectively. On the other hand, the XJHT and AF_Jarso local chicken populations displayed the lowest levels of genetic diversity.

After applying LD pruning, we obtained 0.75 million SNPs to perform population structure analysis. The result of the PCA revealed distinct genetic structures among samples from different geographic regions, with most domestic chickens clustered based on their origin and location (Fig. 1b). Specifically, populations from Southeast Asia (Indonesia, Vietnam, and Thailand) clustered together, while populations from southern China formed another distinct cluster (GD, GX, YN, and Hainan). Populations from southern China were close to those from Southeast Asia. Populations from northern China (HLJ, BJ) clustered near those from central and eastern China (Hubei, Jiangsu). Populations from western China (XJBC and XJHT) clustered with those from Sri_Lanka and Iran. Additionally, populations from Africa clustered together (AF_Jarso and AF_Horro). Admixture results from K = 2 to K = 15 are shown in Additional file 3 Figure S2a. At K = 5, the cross-validation error was the lowest (see Additional file 3 Figure S2b and Fig. 1c) and five distinct ancestral clusters were observed: one cluster included populations from China (excluding domestic chickens from TW); another comprised populations from Southeast Asia; a third cluster included populations from Central Asia, South Asia, and Africa; a fourth cluster consisted of chickens from TW; and the fifth cluster contained chickens from Canada. Notably, chickens from Canada and TW formed two separate clusters, reflecting the diversity in chickens’ genetic backgrounds. Furthermore, populations from western China shared ancestral components with those from South Asia, Central Asia, and Africa, while populations from southern China (excluding domestic chickens from TW) shared genetic components with those from Southeast Asia.

### GEA and functional analyses

**Fig. 2:**
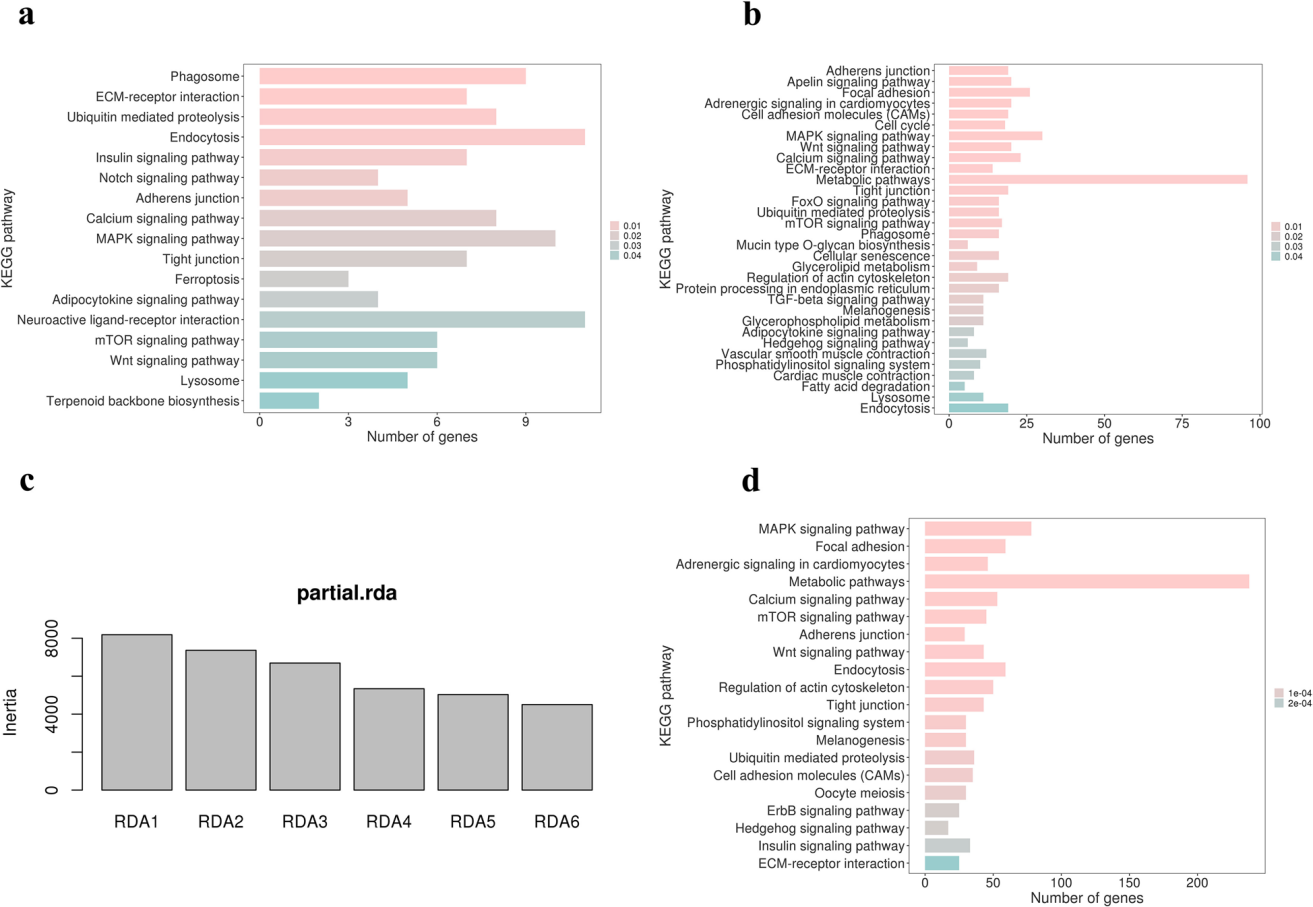
Plot of the significant KEGG pathways. **a** The significant KEGG pathway based on the genes identified by LFMM; **b** The significant KEGG pathway based on the genes identified by individual-based spatial analysis; **c** Variance explained by RDA axes; **d** the top 20 KEGG pathway based on the genes identified by RDA

In this study, we employed three GEA analyses to explore the genetic basis of climate adaptation in domestic chickens. The LFMM identified 1464 significant SNPs and 688 protein-coding genes related to one or more climatic variables (see Additional file 1 Table S6). KEGG analysis showed that these genes were significantly enriched in the phagosome, endocytosis, extracellular matrix (ECM)-receptor interaction, insulin signaling, and adipocytokine signaling pathways (Fig. 2a).

An individual-based spatial analysis conducted using Samβada identified 5552 significant SNPs and 1663 protein-coding genes (see Additional file 1 Table S7). KEGG analysis revealed that the metabolic pathway was the most significantly enriched (Fig. 2b).

The model constructed for the RDA was highly significant (*P*-value = 0.001) and explained about 2.3% of the genetic variation. Each of the six constrained RDA axes explained 10 to 30% of the variance captured by the model (Fig. 2c). We ultimately detected 12,584 significant SNPs that showed extreme loadings (standard deviation > 3) along one or more RDA axes (see Additional file 1 Table S8). From these outliers, 3730 protein-coding genes were annotated. The top 20 KEGG enrichment results further highlighted the importance of the metabolic pathway in climate adaptation of domestic chickens (Fig. 2d).

**Fig. 3:**
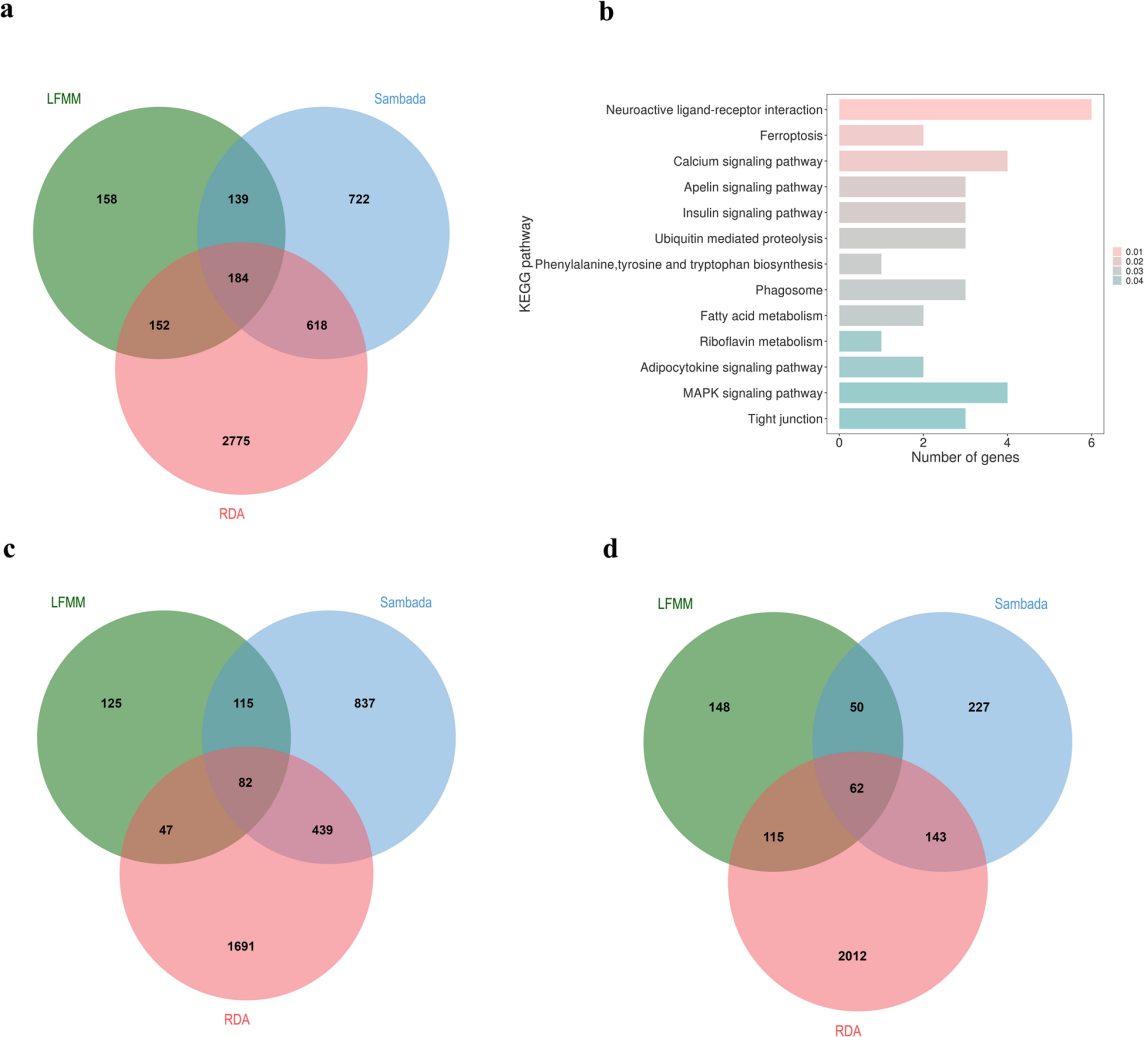
Overlap of genes identified in the different GEA analysis methods. **a** The Venn diagram displaying the overlap of climate-adaptation candidate genes identified by LFMM, individual-based spatial analysis, and RDA. **b** The significant KEGG pathway of the 184 genes identified by both the LFMM, individual-based spatial analysis and RDA; **c** The Venn diagram displaying the overlap of temperature-adaptation candidate genes identified by the LFMM, individual-based spatial analysis, and RDA; **d** The Venn diagram displaying the overlap of precipitation-adaptation candidate genes identified by the LFMM, individual-based spatial analysis, and RDA

By comparing the results from the three methods, we identified 184 protein-coding genes that were detected by all three methods (Fig. 3a and see Additional file 1 Table S9). Notably, 46 of these genes overlapped with QTLs that have been reported to be associated with chicken adaptation, including adaptation to the minimum temperature of the coldest month [28], precipitation in the wettest quarter [28], variation in precipitation across a year [28], tropical climates [12], frigid environments [54], drought environments [54], cold environments [31, 55], hot‑arid environments [56], soil organic carbon [28], and the proportion of cultivated land [28] (see Additional file 1 Table S10). Furthermore, KEGG analysis of the 184 genes revealed significant enrichment in neuroactive ligand-receptor interaction, metabolic, and insulin signaling pathways (Fig. 3b). Among these enriched pathways, the neuroactive ligand-receptor interaction pathway was the most significant, involving the following six genes: *glutamate ionotropic receptor delta type subunit 1* (*GRID1*), *gamma-aminobutyric acid type A receptor rho2 subunit* (*GABRR2*), *glutamate ionotropic receptor kainate type subunit 4* (*GRIK4*), *thyroid hormone receptor* (*THRA*), *complement component 3* (*C3*), and *thyroid stimulating hormone receptor* (*TSHR*).

Subsequently, we categorized the climatic variables into those related to temperature and precipitation and separately analyzed the GEA results for temperature-related and precipitation-related climatic variables. Ultimately, we identified 82 genes associated with temperature-related climatic variables, which we propose may be linked to temperature adaptation in domestic chickens (Fig. 3c and see Additional file 1 Table S11). Of these 82 genes, seven are involved in the regulation of immune function, four are associated with lipid biosynthesis and storage, and one is related to the regulation of body fluid levels. Notably, two genes, *crumbs cell polarity complex component 1* (*CRB1*) and *zinc finger protein 536* (*ZNF536*), have been shown to play significant roles in cold adaptation in animals [31, 65]. In addition, we identified 62 genes associated with precipitation-related climatic variables, which we suggest may contribute to precipitation adaptation in domestic chickens (Fig. 3d and see Additional file 1 Table S12). These genes are involved in regulation of the immune system and play roles in angiogenesis, lipid transport and metabolism, and development of the urogenital system. Both the 82 and the 62 genes described above are part of the 184 genes that were initially identified using the 19 climatic variables.

### Candidate gene analysis

**Fig. 4:**
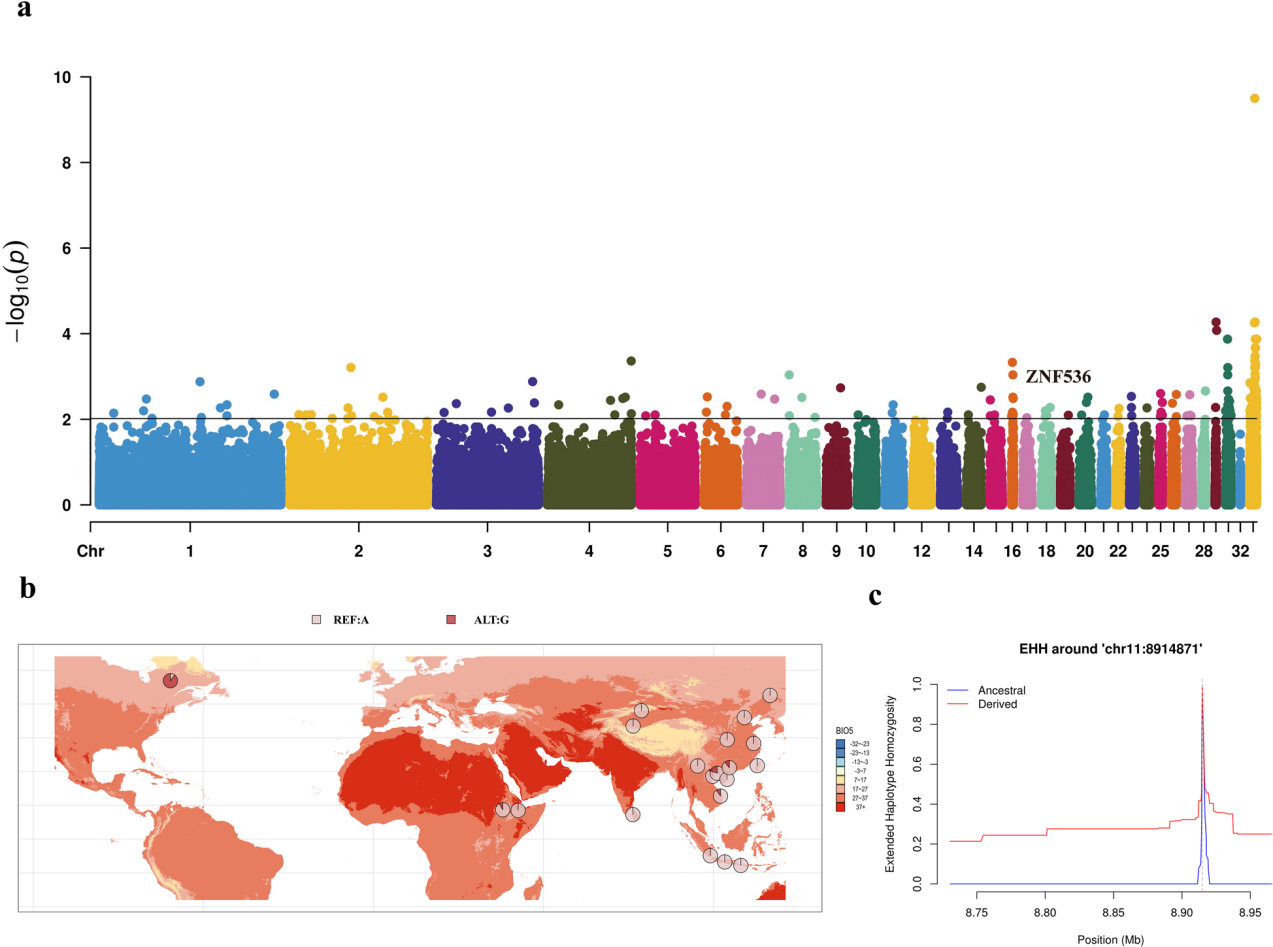
Association analysis results for the *ZNF536* gene. **a** Manhattan plot of LFMM results for variants associated with BIO5. The horizontal black line represents the significance threshold (FDR correction, adjusted *P* = 0.01) with the *ZNF536* gene labeled; **b** Allele frequency distribution of a SNP (chr11: 8,914,871 bp) within *ZNF536*. Colors on the map represent the variations of BIO5 across different regions; **c** Diagram of EHH result for chr11: 8,914,871 bp within *ZNF536* across all chicken populations involved in this study

By comparing the allele frequency distribution patterns of SNPs within the 82 genes associated with temperature adaptation and the 62 genes linked to precipitation adaptation across different chicken populations, we detected five SNPs with distinct geographic distribution preferences. Among these five SNPs, three are located in the intronic regions of *ZNF536* and *ENSGALG00000049158*, both of which are linked to the maximum temperature of warmest month (BIO5). The allele frequency distributions of these three intronic variants display fixed patterns in the chickens from Canada (Fig. 4 and see Additional file 4 Figure S3). For example, within the *ZNF536* gene, we identified one SNP (chr11: 8,914,871 bp) for which the mutant allele G exhibited a higher frequency in Canada chickens compared to other populations (Fig. 4b). We also compared the EHH patterns between haplotypes carrying the A or G alleles of this SNP. The results indicated that the haplotype with the derived allele (G) exhibited a slower decrease in EHH than the haplotype with the ancestral allele (A), suggesting positive selection at this locus in Canada chickens (Fig. 4c). Similar results were obtained for the two SNPs within *ENSGALG00000049158* (see Additional file 4 Figure S3). These findings suggest that *ZNF536* and *ENSGALG00000049158* may play significant roles in the local adaptation of chickens in Canada to temperature increases during the warmest month.

**Fig. 5:**
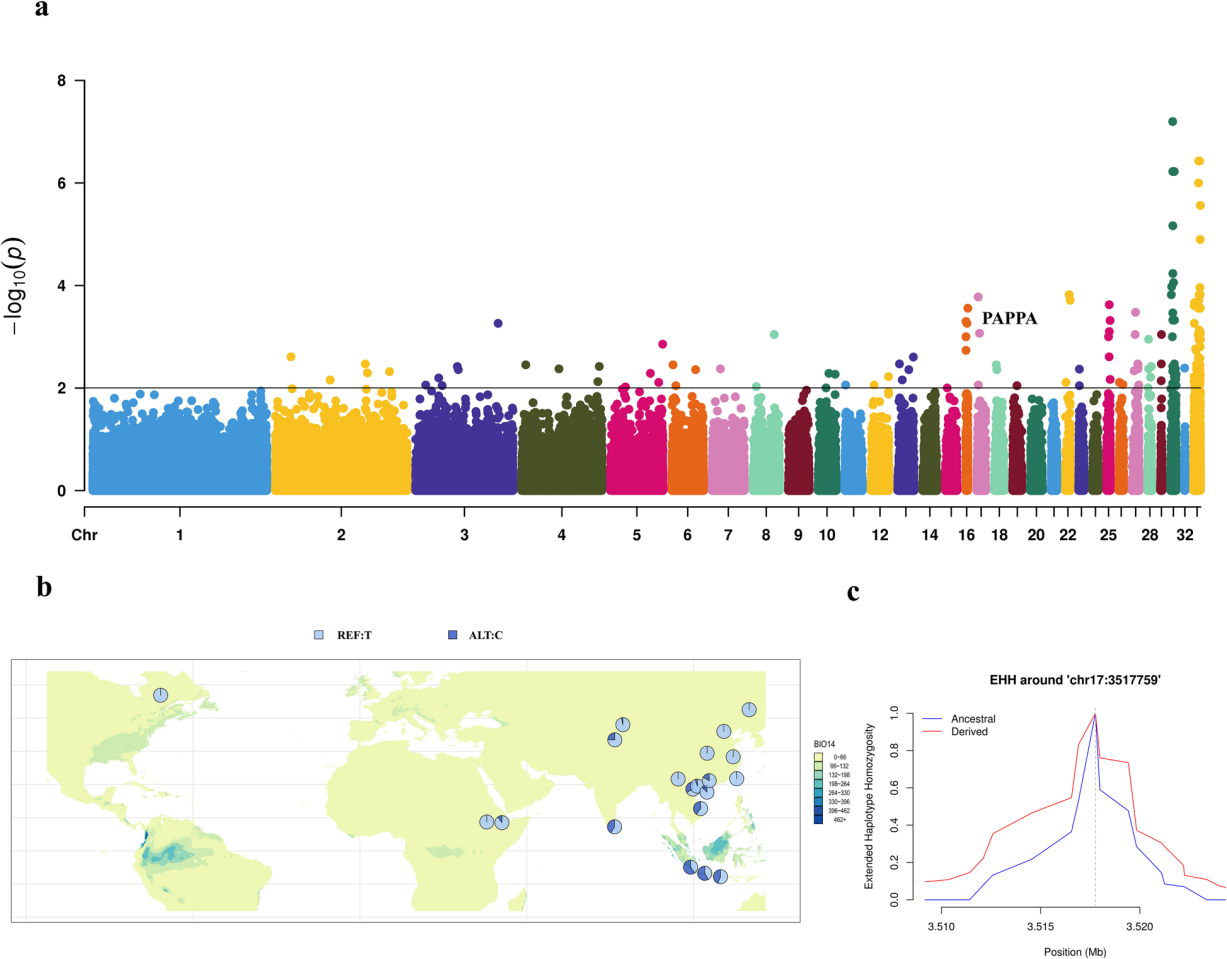
Association analysis results for the *PAPPA* gene. **a** Manhattan plot of LFMM results for variants associated with BIO14. The horizontal black line represents the significance threshold (FDR correction, adjusted *P* = 0.01) with the *PAPPA* gene labeled; **b** Allele frequency distribution of a SNP (chr17: 3,517,759 bp) within *PAPPA*. Colors on the map represent the variations of BIO14 across different regions; **c** Diagram of EHH result for chr17: 3,517,759 bp within *PAPPA* across all chicken populations involved in this study

The other two variants with distinct geographic distribution preferences are located in the intronic regions of *pappalysin 1* (*PAPPA*) and *euchromatic histone lysine methyltransferase 1* (*EHMT1*), both of which were found to be associated with precipitation adaptation. The allele frequency distributions of these two intronic variants exhibit distinct geographic differences (Fig. 5 and see Additional file 5 Figure S4). Within *PAPPA*, we identified one SNP (chr17: 3,517,759 bp) for which the mutant allele C was predominantly distributed in areas with high precipitation during the driest month, while the T allele was largely fixed in regions with low rainfall (Fig. 5b). EHH analysis indicated positive selection at this locus, favoring the haplotype carrying the derived allele (C), which exhibited a slower decrease in EHH compared to the haplotype with the ancestral allele (T) (Fig. 5c). These results suggest a potential link between *PAPPA* and drought adaptation in domestic chickens. Within *EHMT1*, we identified another SNP (chr17: 2,355,759 bp) for which the mutant allele A was more common in areas with high seasonality in precipitation, while the G allele was distributed in areas with low seasonality (see Additional file 5 Figure S4b). EHH analysis suggested strong selection at this locus (see Additional file 5 Figure S4c). These findings indicate that *EHMT1* may play a key role in the adaptation of domestic chickens to rapid changes in water availability.

## Discussion

As the global population grows and human activities have expanded, the scope of animal farming has also increased, with a growing number of livestock being raised in challenging environmental conditions. As a result, the risks associated with extreme environmental stresses have become more pronounced. These stresses not only decrease animal welfare but also present significant challenges for livestock production and development. Therefore, investigating the mechanisms underlying climate adaptation in livestock species, such as domestic chickens, is necessary to improve animal welfare and enhance the associated socioeconomic benefits.

In the present study, we explored the mechanisms of climate adaptation in a sample of 199 indigenous domestic chickens from diverse environments globally. Population structure analysis revealed that indigenous chicken populations from geographically adjacent regions shared similar genetic backgrounds (populations from China; populations from southern China and Southeast Asia; and populations from western China, South Asia, Central Asia, and Africa). This structuring may, to some extent, reflect the differential exchange of genetic materials among chicken populations in neighboring regions, consistent with findings from previous studies [66]. We then conducted GEA analysis, including LFMM, individual-based spatial analysis, and RDA. Unlike conventional selection signature analysis, this approach provides a foundation for identifying genetic variants associated with specific climatic factors [23]. Additionally, by integrating multiple methods to screen for the same candidate SNPs and genes, we were able to verify the reliability of our results and reduce the potential risk of false positives [46].

Notably, we performed two separate analyses to investigate the environmental adaptation of chickens. First, we directly integrated the GEA results for 19 climatic variables without categorizing them by type, which allowed us to explore the mechanisms of chicken adaptation driven by the combined effects of temperature and precipitation. Next, we categorized the climatic variables into temperature-related and precipitation-related factors, and then independently analyzed the GEA results for each category. This method enabled us to identify genes specifically associated with temperature adaptation and those related to precipitation adaptation, providing new insights into the mechanisms underlying chicken adaptation to different environmental factors.

By integrating the GEA results for the 19 climatic variables, we identified 184 candidate genes. Further KEGG analysis of these genes revealed significant enrichment of the neuroactive ligand-receptor interaction pathway, which is involved in the nervous system. This pathway has been shown to be related to heat tolerance in cattle, Peking ducks, and the oriental river prawn *Macrobrachium nipponense* [67–70]. Notably, the *TSHR* gene was among the six genes associated with this pathway. This gene encodes a receptor for thyroid-stimulating hormone, a key regulator of thyroid function [70]. It is involved in reproduction, energy balance, metabolism, and thermoregulation [70–72]. Numerous studies have demonstrated its role in heat tolerance in chickens [18, 56, 73, 74]. In the present study, *TSHR* was identified by LFMM and Samβada to be associated with precipitation-related climatic variables, and by RDA and Samβada to be associated with temperature-related climatic variables. Details are provided in Additional File 1 Tables S6-S8. Thus, when categorizing climatic variables and screening genes that were identified by all three methods to associated with either temperature-related or precipitation-related variables, *TSHR* was not detected. However, when screening genes without categorizing variable types, *TSHR* was identified. This may be the result of differences in the three GEA analysis methods employed. Nevertheless, our findings further support the importance of *TSHR* in climate adaptation in chickens, as it is associated with both temperature adaptation and precipitation adaptation. We further analyzed the allele frequency distribution patterns of SNPs within the *TSHR* gene across different chicken populations and detected two missense variants (chr5: 41,019,556 bp and chr5: 41,020,238 bp) with distinct geographic distribution preferences. Specifically, the mutant alleles of these two SNPs are primarily distributed in regions with high annual precipitation and high precipitation during the wettest quarter. EHH analysis provided evidence of positive selection at these two loci (Additional files 6, 7, 8, 9). This study further highlights the critical role of *TSHR* in chicken adaptation. Thus, exploring the function of *TSHR* in climate adaptation in future research remains crucial.

Next, by separately analyzing the GEA results for temperature-related and precipitation-related climatic variables, we identified 82 genes associated with temperature adaptation and 62 genes associated with precipitation adaptation. We further investigated the functions of these 82 and 62 genes and found that both groups contained immune-related genes of which two have been reported to be associated with environmental adaptation in animals: *WNK1* and *NOTCH2*. The *wnk lysine deficient protein kinase 1* (*WNK1*) may regulate migration and adhesion of B cells, which is crucial for humoral immune responses [75]. Liu et al. identified *WNK1* as a candidate gene for cold tolerance in Chinese indigenous cattle [76]. The *notch 2* (*NOTCH2*) plays a critical role in the body’s immune homeostasis [77] and has been reported to be related to high-altitude adaptation in animals such as the high-altitude Leopard and Tibetan chicken [78]. Many studies have demonstrated the role of immune mechanisms in climate adaptation across various species, including cattle, sheep, and chickens [12, 28, 60, 79, 80]. Our findings further support the notion that immune-related genes may play a crucial role in climate adaptation in chickens.

By further analyzing the allele frequency distribution patterns of SNPs within 82 genes associated with temperature adaptation and 62 genes associated with precipitation adaptation, we identified four genes that may play significant roles in climate adaptation in chickens: *ZNF536*, *ENSGALG00000049158*, *PAPPA*, and *EHMT1*. Among these, *ZNF536* and *ENSGALG00000049158* were associated with the maximum temperature of the warmest month, suggesting their potential contribution to the local adaptation of chickens in Canada to temperature increases during the warmest month. *ZNF536* is involved in the development of forebrain neurons related to social behavior and stress responses [81, 82] and has been reported to be related to cold adaptation in Canada chickens [31]. Notably, the Canada chickens used in the present study were derived from that research. Taken together, our findings support the importance of *ZNF536* in the temperature adaptation of Canada chickens, although the precise mechanisms underlying its function remain to be elucidated. Although the function of *ENSGALG00000049158* is not yet fully understood, our findings provide preliminary insights into its potential role.

The *PAPPA* gene has been identified to be associated with drought adaptation, while *EHMT1* has been linked to adaptation to rapid changes in water availability. Both these two genes are involved in the regulation of glucose homeostasis [83, 84], a biological process crucial for climatic adaptation in animals [85]. Our study provides the first evidence that *PAPPA* and *EHMT1* may be related to precipitation adaptation in chickens.

## Conclusions

Using landscape genomics, we provide new insights into the genetic mechanisms underlying climate adaptation in domestic chickens. By employing three different GEA analyses, we identified a set of candidate genes associated with climate adaptation. Our findings indicate that the *TSHR* gene has multiple roles in climate adaptation, while immune-related genes also contribute to this adaptation in chickens. We identified four genes, *ZNF536*, *ENSGALG00000049158*, *PAPPA*, and *EHMT1*, that may play significant roles in chickens’ adaptation driven by diverse climatic factors. However, further research is required to validate the function of these genes and their corresponding mutations. Our results deepen the understanding of the genetic mechanisms of climate-driven adaptation in domestic chickens and other livestock species, providing a foundation for the use of molecular markers in breeding populations with varying degrees of adaptability.

## Supplementary Information

**Additional file 1: Table S1. **Summary of samples included in this study. **Table S2.** Summary of climatic variables included in this study. **Table S3.** Summary of genome sequencing and mapping statistic. **Table S4.** Details of SNPs' repartition across the autosomal chromosomes. **Table S5.** Within-population genetic diversity. **Table S6.** 1464 candidate SNPs associated with climatic variables as identified by the LFMM approach. **Table S7.** 5552 candidate SNPs associated with climatic variables as identified by the Samβada. **Table S8.** 12,584 candidate SNPs associated with climatic variables as identified by the RDA. **Table S9.** 184 genes identified by the LFMM, individual-based spatial analysis, and RDA. **Table S10.** The overlap of candidante genes with reported QTLs related to the adaptation of chickens. **Table S11.** 82 genes associated with temperature adaptation identified by the LFMM, individual-based spatial analysis, and RDA. **Table S12.** 62 genes associated with precipitation adaptation identified by the LFMM, individual-based spatial analysis, and RDA.

**Additional file 2: Figure S1.** The results of ranked importance and correlation between the 19 climatic variables. (a) R2-weighted ranked importance of climatic variables from gradient forest analysis. Climatic variables related to temperature are represented in red, while variables related to precipitation are represented in blue; (b) Plot of the correlation between the 19 climatic variables.

**Additional file 3: Figure S2.** Admixture results from K = 2 to K = 15. (a) Plot of the admixture results from K = 2 to K = 15; (b) Plot of the cross-validation error from K = 2 to K = 15.

**Additional file 4: Figure S3.** Association analysis results for the *ENSGALG00000049158* gene. (a) Manhattan plot of LFMM results for variants associated with BIO5. The horizontal black line represents the significance threshold (FDR correction, adjusted *P* = 0.01) with the *ENSGALG00000049158* gene labeled; (b), (d) Allele frequency distributions of two SNPs (chr33: 728,004 bp and chr33: 817,465 bp) within *ENSGALG00000049158*. Colors on the map represent the variations of BIO5 across different regions; (c), (e) Diagram of EHH result for chr33: 728,004 bp and chr33: 817,465 bp within *ENSGALG00000049158* across all chicken populations involved in this study.

**Additional file 5: Figure S4.** Association analysis results for the *EHMT1* gene. (a) Manhattan plot of LFMM results for variants associated with BIO15. The horizontal black line represents the significance threshold (FDR correction, adjusted *P* = 0.01) with the *EHMT1* gene labeled; (b) Allele frequency distribution of chr17: 2,355,759 bp within *EHMT1*. Colors on the map represent the variations of BIO15 across different regions; (c) Diagram of EHH result for chr17: 2,355,759 bp within *EHMT1* across all chicken populations involved in this study.

**Additional file 6: Figure S5. **Association analysis results for the *TSHR* gene. (a) Manhattan plot of LFMM results for variants associated with BIO12. The horizontal black line represents the significance threshold (FDR correction, adjusted *P* = 0.01) with the *TSHR* gene labeled; (b) Allele frequency distribution of chr5: 41,019,556 bp within *TSHR*. Colors on the map represent the variations of BIO12 across different regions; (c) Diagram of EHH result for chr5: 41,019,556 bp within *TSHR *across all chicken populations involved in this study.

**Additional file 7: Figure S6.** Association analysis results for the *TSHR* gene. (a) Manhattan plot of LFMM results for variants associated with BIO12. The horizontal black line represents the significance threshold (FDR correction, adjusted *P* = 0.01) with the *TSHR* gene labeled; (b) Allele frequency distribution of chr5: 41,020,238 bp within *TSHR*. Colors on the map represent the variations of BIO12 across different regions; (c) Diagram of EHH result for chr5: 41,020,238 bp within *TSHR *across all chicken populations involved in this study.

**Additional file 8: Figure S7.** Association analysis results for the *TSHR* gene. (a) Manhattan plot of LFMM results for variants associated with BIO16. The horizontal black line represents the significance threshold (FDR correction, adjusted *P* = 0.01) with the *TSHR* gene labeled; (b) Allele frequency distribution of chr5: 41,019,556 bp within *TSHR*. Colors on the map represent the variations of BIO16 across different regions; (c) Diagram of EHH result for chr5: 41,019,556 bp within *TSHR *across all chicken populations involved in this study.

**Additional file 9: Figure S8.** Association analysis results for the *TSHR* gene. (a) Manhattan plot of LFMM results for variants associated with BIO16. The horizontal black line represents the significance threshold (FDR correction, adjusted *P* = 0.01) with the *TSHR* gene labeled; (b) Allele frequency distribution of chr5: 41,020,238 bp within *TSHR*. Colors on the map represent the variations of BIO16 across different regions; (c) Diagram of EHH result for chr5: 41,020,238 bp within *TSHR *across all chicken populations involved in this study.

**Additional file 10: Figure S9.** Plot of the correlation among the 19 climatic variables in different areas. (a) Plot of the correlation among the 19 climatic variables in Northern China; (b) Plot of the correlation among the 19 climatic variables in Western China; (c) Plot of the correlation among the 19 climatic variables in Southern China; (d) Plot of the correlation among the 19 climatic variables in South Asia; (e) Plot of the correlation among the 19 climatic variables in Southeast Asia.

## Data Availability

The data generated in the current study are available under the NCBI accession numbers of PRJNA871052 and PRJNA724749.
