# Supplementary figures and images for: Genome-environment association analysis reveals climate-driven adaptation of chickens
Source: Genet Sel Evol. 2025 Jul 22;57:43. doi: 10.1186/s12711-025-00989-9 (PMC12281946; doi:10.1186/s12711-025-00989-9)

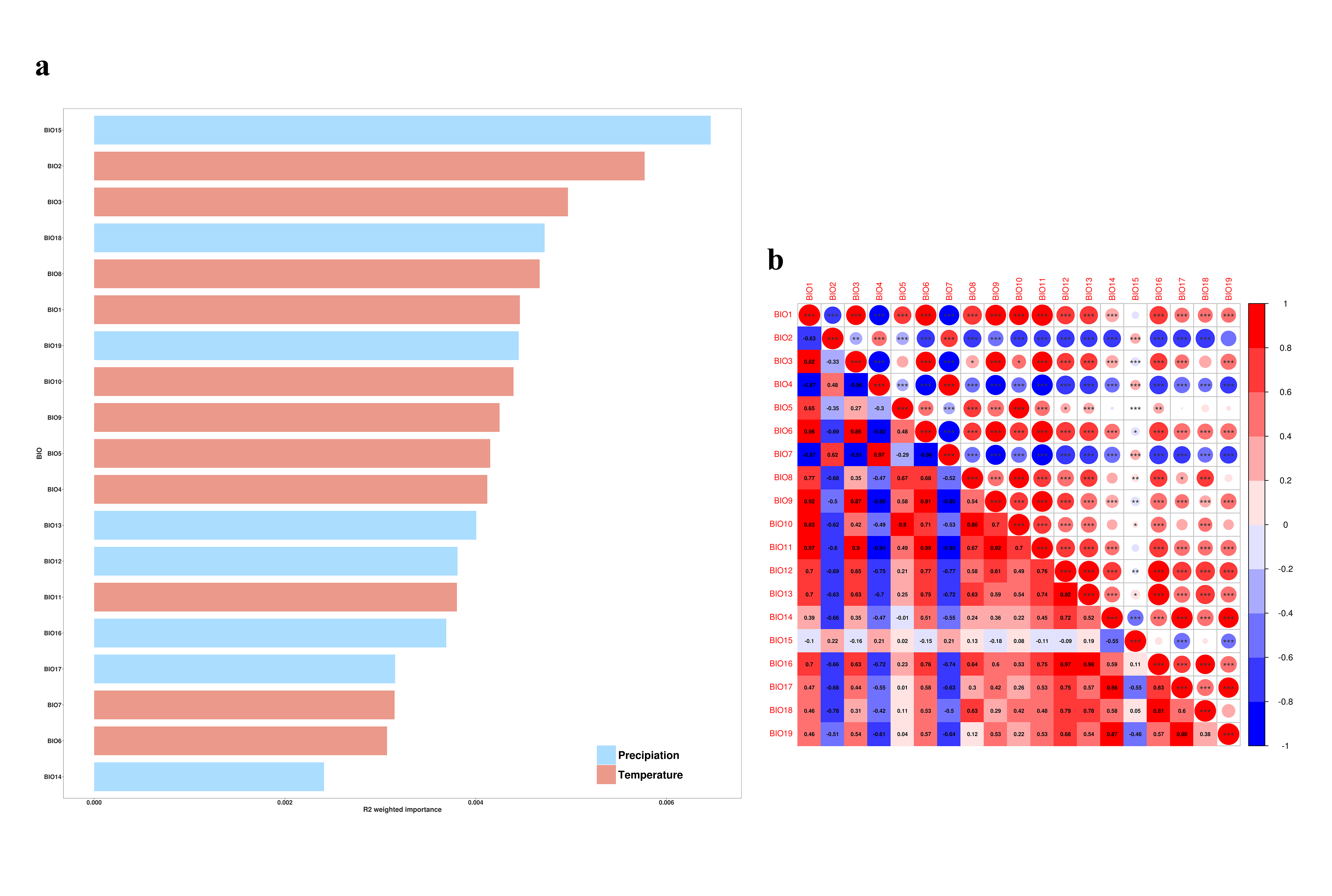

Supplement: Supplementary file 2 — Additional file 2: Figure S1. The results of ranked importance and correlation between the 19 climatic variables. (a) R2-weighted ranked importance of climatic variables from gradient forest analysis. Climatic variables related to temperature are represented in red, while variables related to precipitation are represented in blue; (b) Plot of the correlation between the 19 climatic variables. [file 12711_2025_989_MOESM2_ESM.tif]

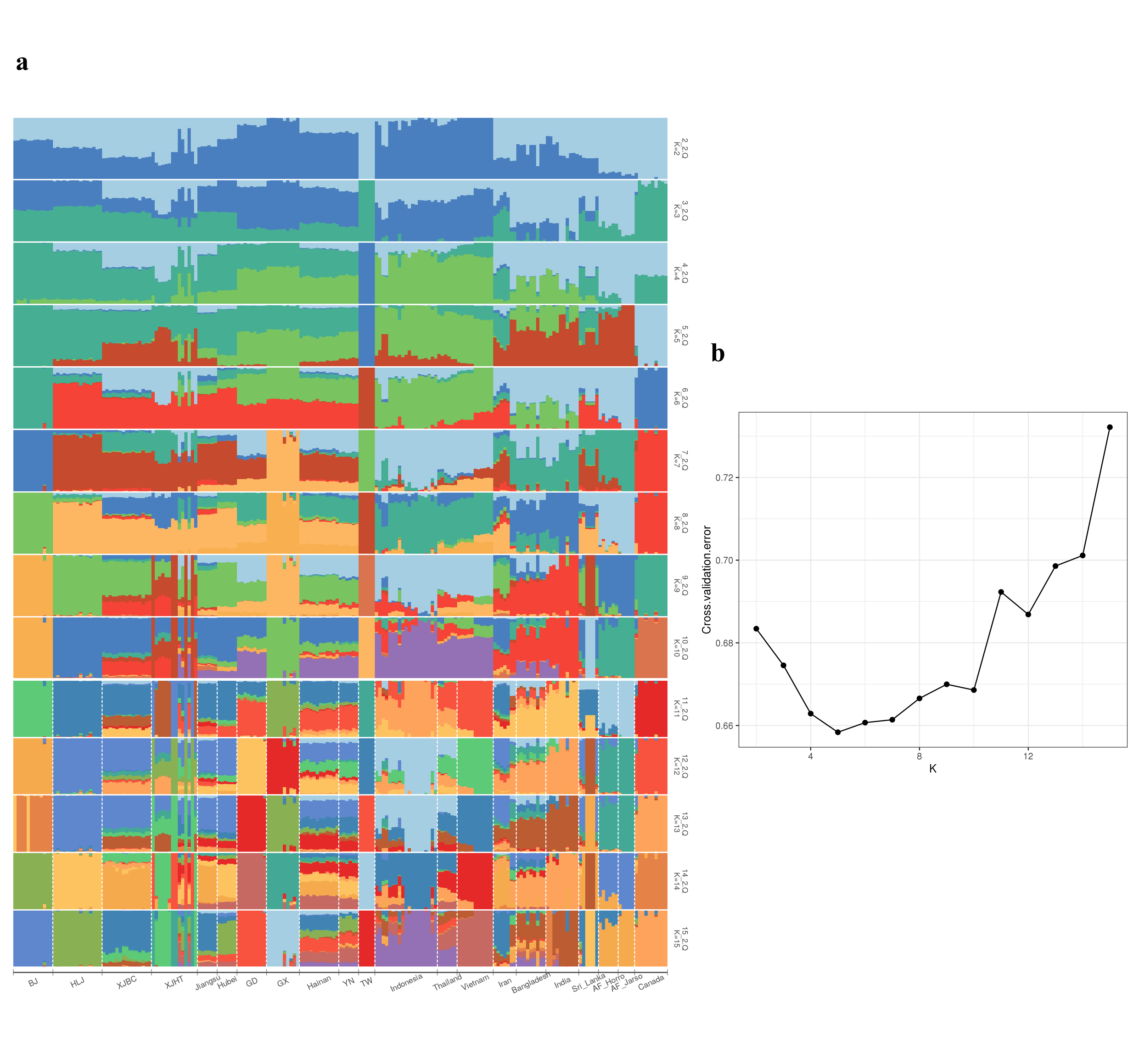

Supplement: Supplementary file 3 — Additional file 3: Figure S2. Admixture results from K = 2 to K = 15. (a) Plot of the admixture results from K = 2 to K = 15; (b) Plot of the cross-validation error from K = 2 to K = 15. [file 12711_2025_989_MOESM3_ESM.tif]

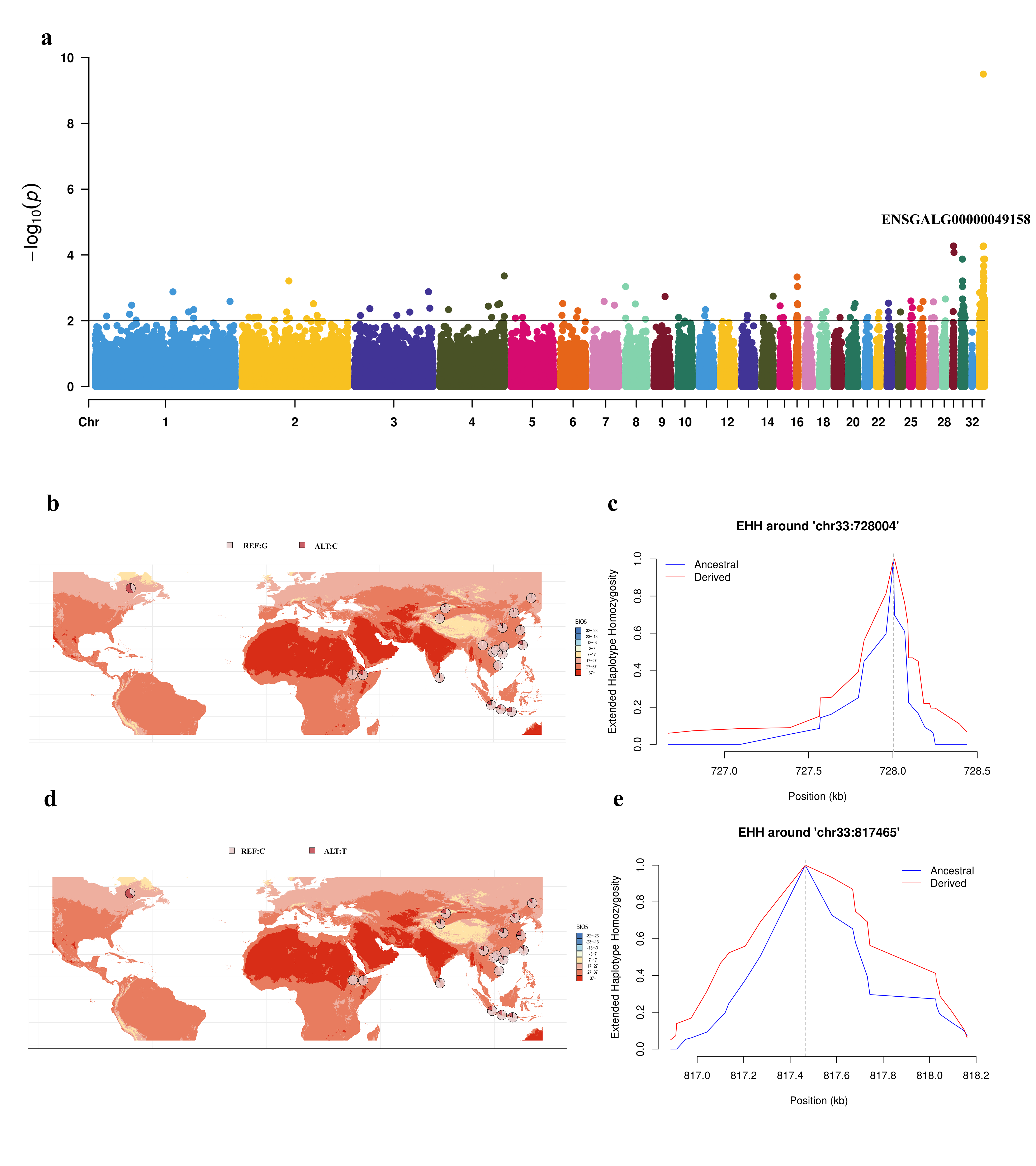

Supplement: Supplementary file 4 — Additional file 4: Figure S3. Association analysis results for the ENSGALG00000049158 gene. (a) Manhattan plot of LFMM results for variants associated with BIO5. The horizontal black line represents the significance threshold (FDR correction, adjusted P = 0.01) with the ENSGALG00000049158 gene labeled; (b), (d) Allele frequency distributions of two SNPs (chr33: 728,004 bp and chr33: 817,465 bp) within ENSGALG00000049158. Colors on the map represent the variations of BIO5 across different regions; (c), (e) Diagram of EHH result for chr33: 728,004 bp and chr33: 817,465 bp within ENSGALG00000049158 across all chicken populations involved in this study. [file 12711_2025_989_MOESM4_ESM.tif]

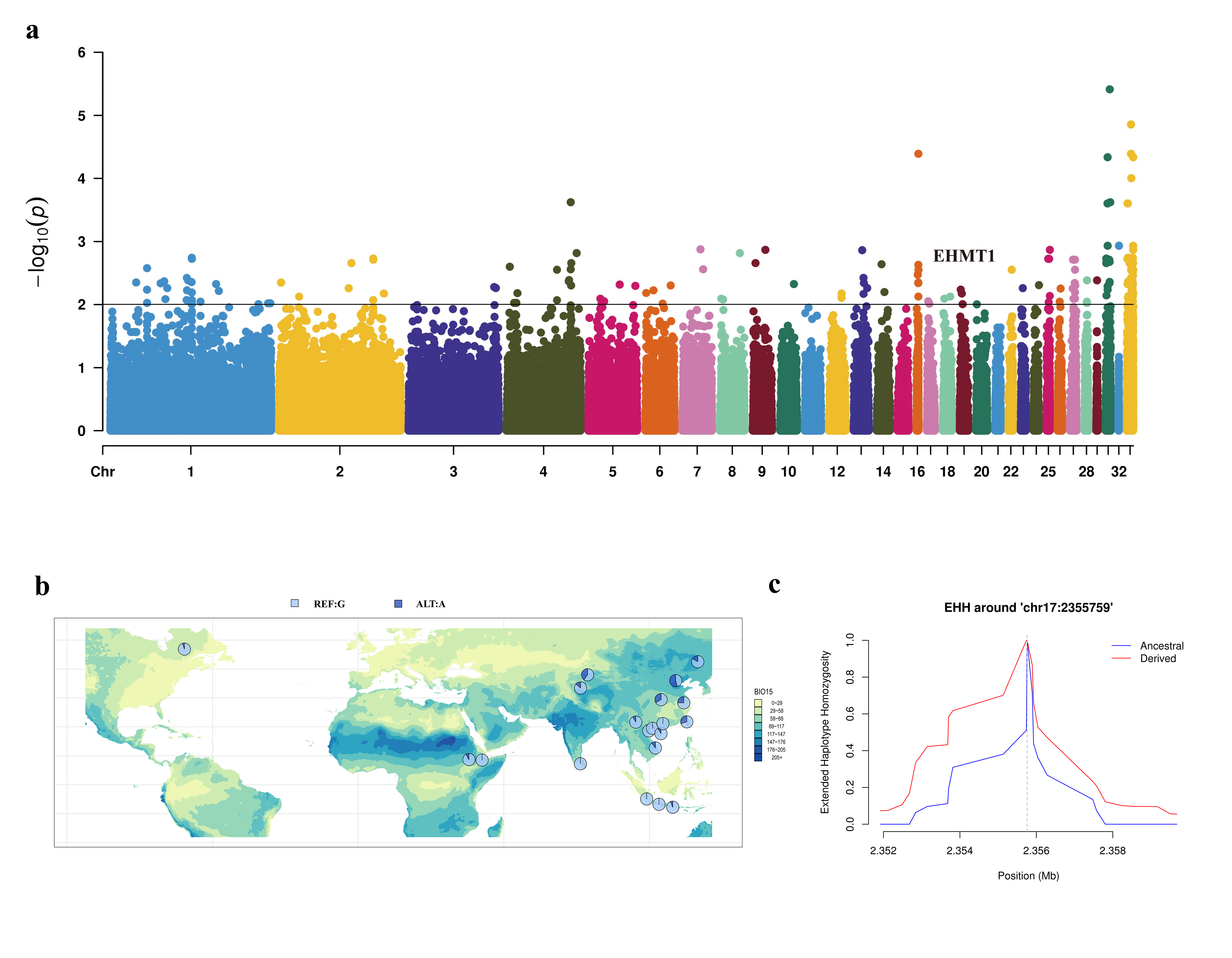

Supplement: Supplementary file 5 — Additional file 5: Figure S4. Association analysis results for the EHMT1 gene. (a) Manhattan plot of LFMM results for variants associated with BIO15. The horizontal black line represents the significance threshold (FDR correction, adjusted P = 0.01) with the EHMT1 gene labeled; (b) Allele frequency distribution of chr17: 2,355,759 bp within EHMT1. Colors on the map represent the variations of BIO15 across different regions; (c) Diagram of EHH result for chr17: 2,355,759 bp within EHMT1 across all chicken populations involved in this study. [file 12711_2025_989_MOESM5_ESM.tif]

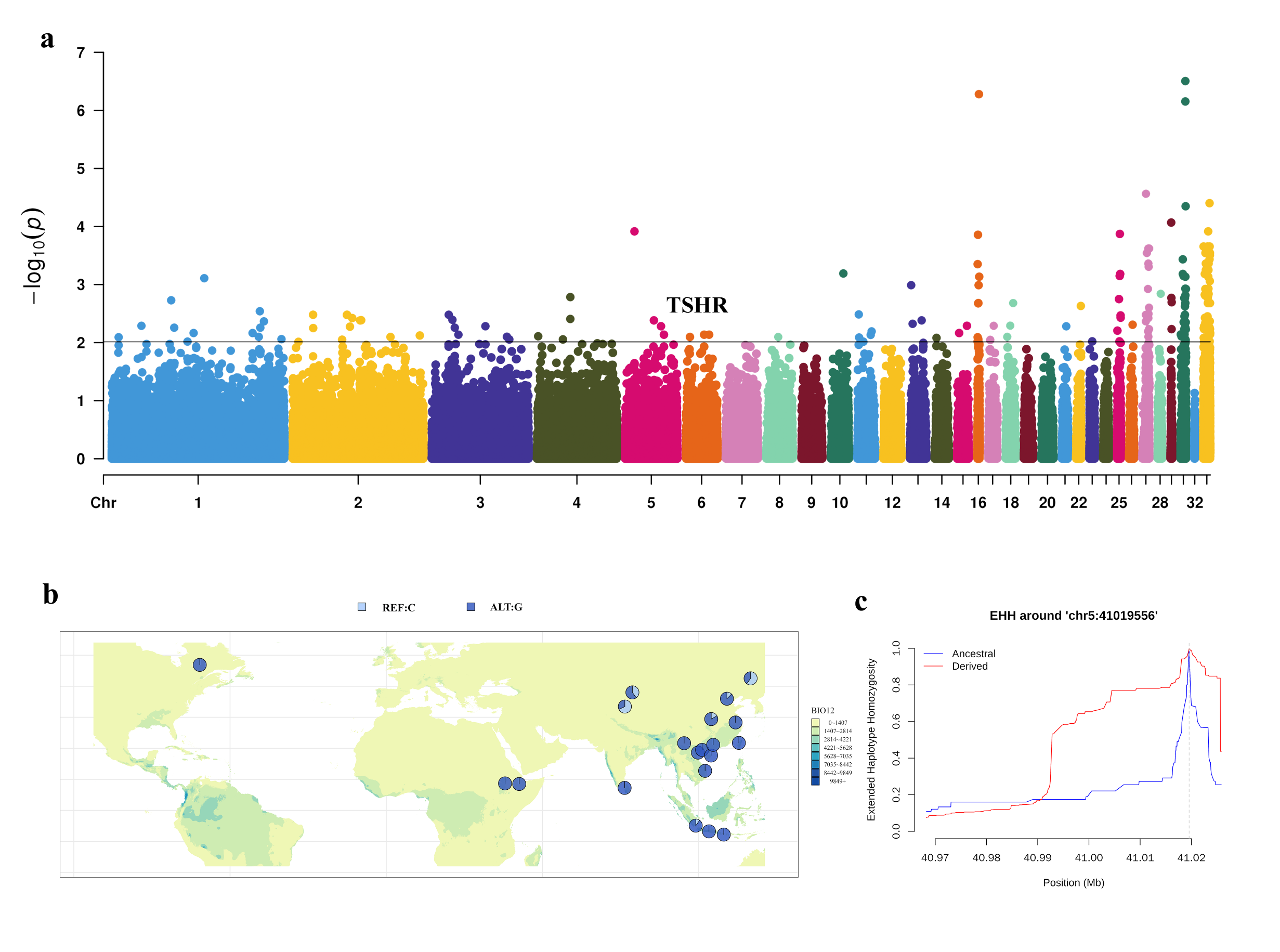

Supplement: Supplementary file 6 — Additional file 6: Figure S5. Association analysis results for the TSHR gene. (a) Manhattan plot of LFMM results for variants associated with BIO12. The horizontal black line represents the significance threshold (FDR correction, adjusted P = 0.01) with the TSHR gene labeled; (b) Allele frequency distribution of chr5: 41,019,556 bp within TSHR. Colors on the map represent the variations of BIO12 across different regions; (c) Diagram of EHH result for chr5: 41,019,556 bp within TSHR across all chicken populations involved in this study. [file 12711_2025_989_MOESM6_ESM.tif]

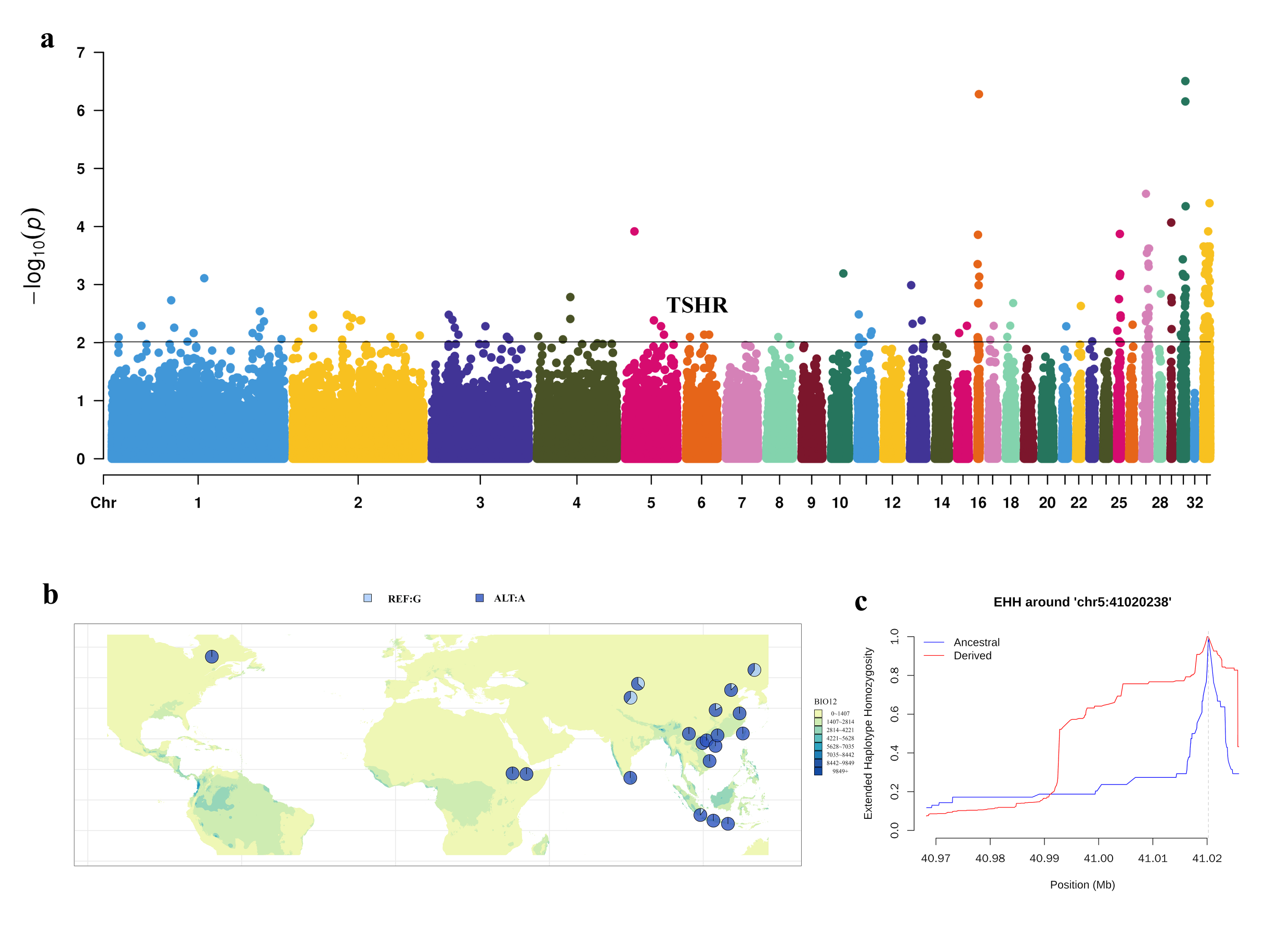

Supplement: Supplementary file 7 — Additional file 7: Figure S6. Association analysis results for the TSHR gene. (a) Manhattan plot of LFMM results for variants associated with BIO12. The horizontal black line represents the significance threshold (FDR correction, adjusted P = 0.01) with the TSHR gene labeled; (b) Allele frequency distribution of chr5: 41,020,238 bp within TSHR. Colors on the map represent the variations of BIO12 across different regions; (c) Diagram of EHH result for chr5: 41,020,238 bp within TSHR across all chicken populations involved in this study. [file 12711_2025_989_MOESM7_ESM.tif]

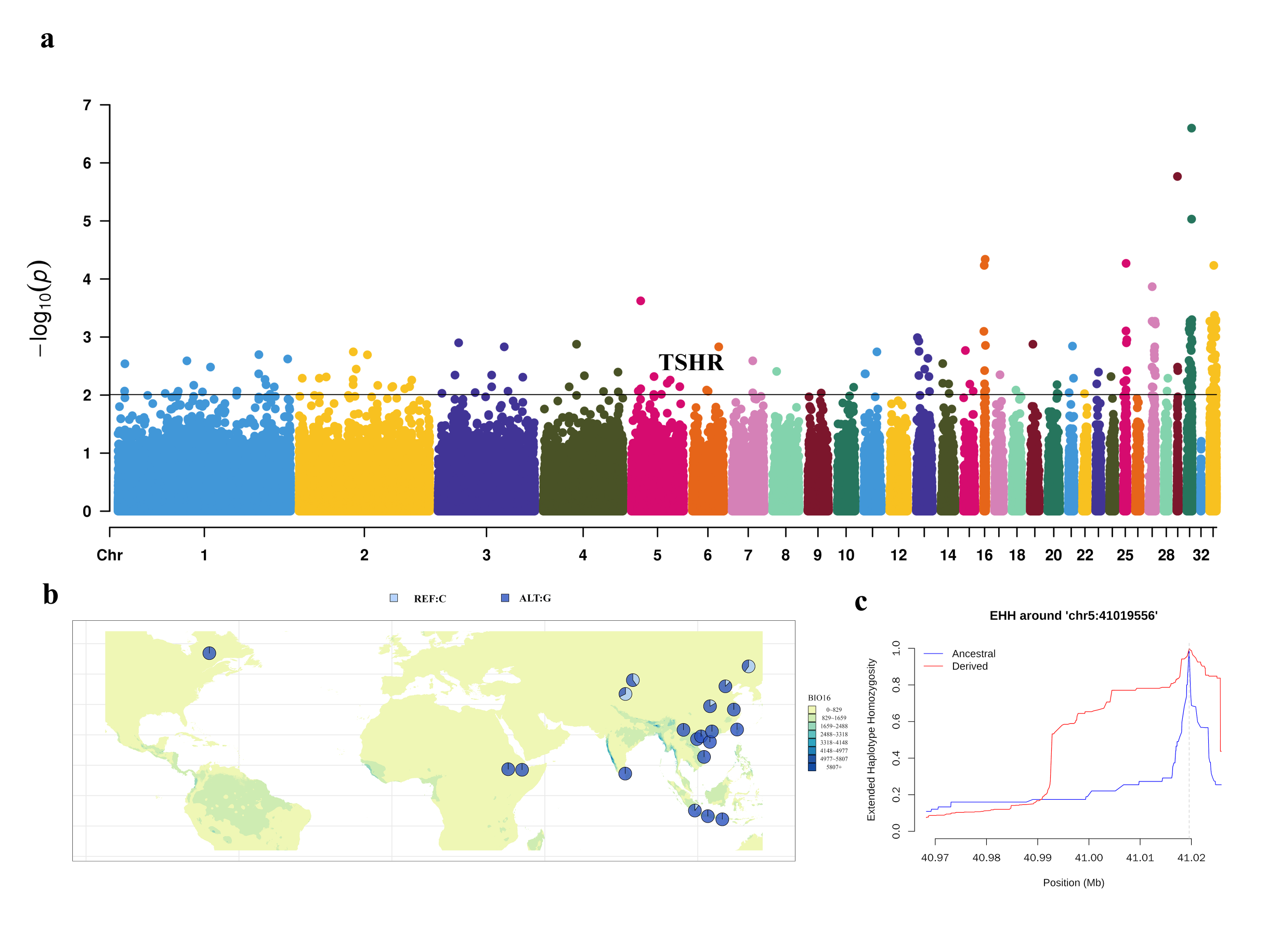

Supplement: Supplementary file 8 — Additional file 8: Figure S7. Association analysis results for the TSHR gene. (a) Manhattan plot of LFMM results for variants associated with BIO16. The horizontal black line represents the significance threshold (FDR correction, adjusted P = 0.01) with the TSHR gene labeled; (b) Allele frequency distribution of chr5: 41,019,556 bp within TSHR. Colors on the map represent the variations of BIO16 across different regions; (c) Diagram of EHH result for chr5: 41,019,556 bp within TSHR across all chicken populations involved in this study. [file 12711_2025_989_MOESM8_ESM.tif]

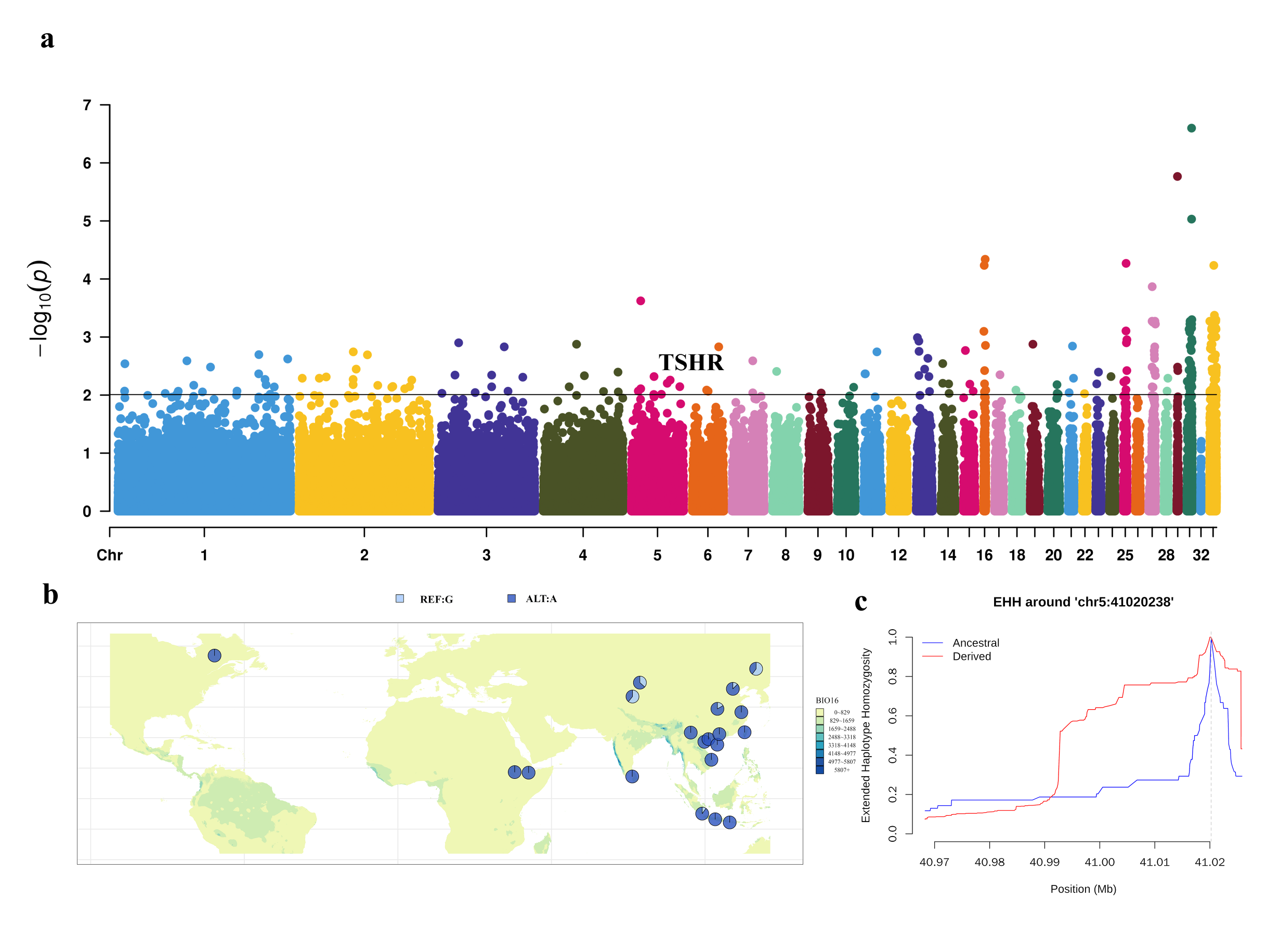

Supplement: Supplementary file 9 — Additional file 9: Figure S8. Association analysis results for the TSHR gene. (a) Manhattan plot of LFMM results for variants associated with BIO16. The horizontal black line represents the significance threshold (FDR correction, adjusted P = 0.01) with the TSHR gene labeled; (b) Allele frequency distribution of chr5: 41,020,238 bp within TSHR. Colors on the map represent the variations of BIO16 across different regions; (c) Diagram of EHH result for chr5: 41,020,238 bp within TSHR across all chicken populations involved in this study. [file 12711_2025_989_MOESM9_ESM.tif]

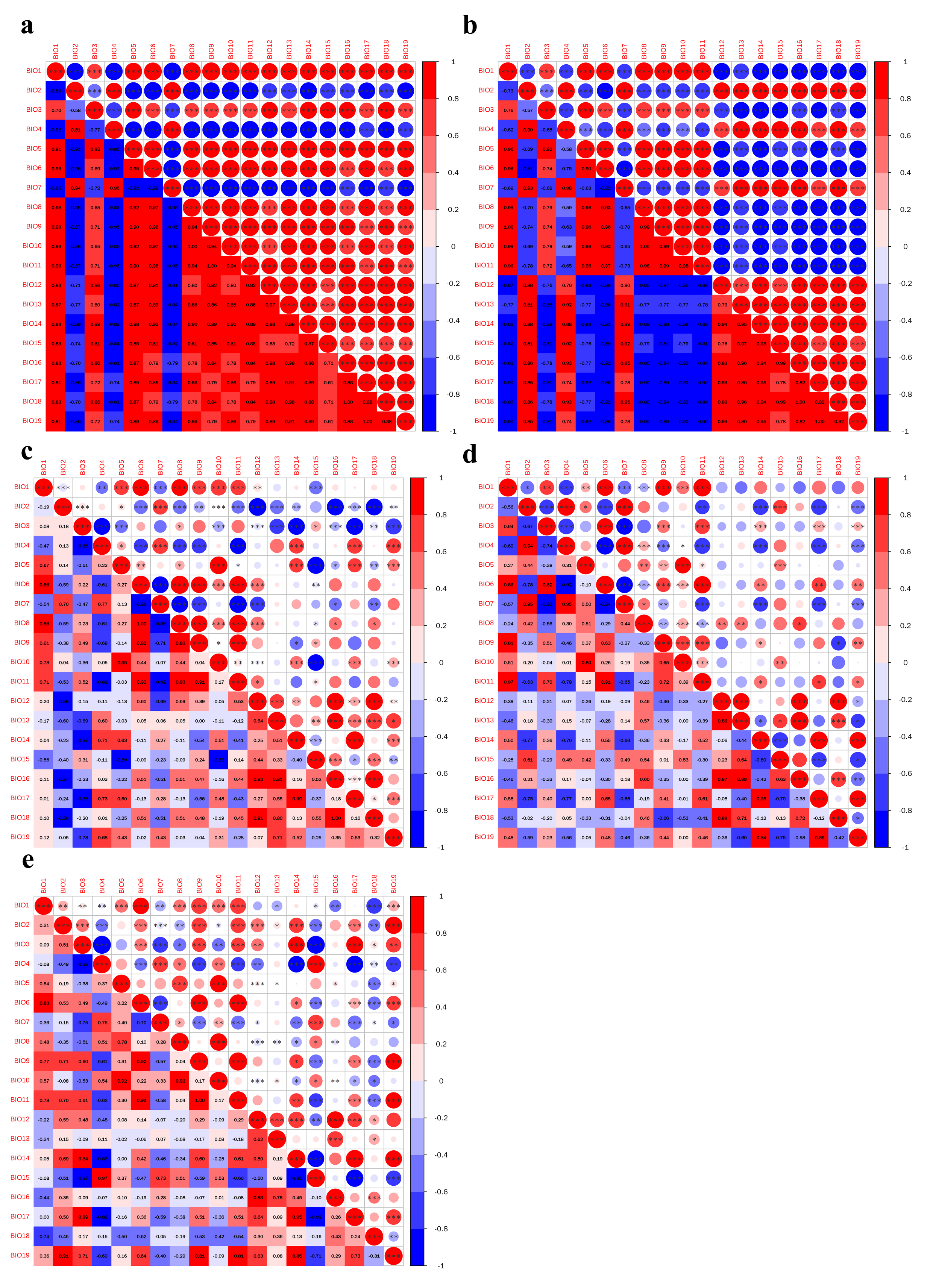

Supplement: Supplementary file 10 — Additional file 10: Figure S9. Plot of the correlation among the 19 climatic variables in different areas. (a) Plot of the correlation among the 19 climatic variables in Northern China; (b) Plot of the correlation among the 19 climatic variables in Western China; (c) Plot of the correlation among the 19 climatic variables in Southern China; (d) Plot of the correlation among the 19 climatic variables in South Asia; (e) Plot of the correlation among the 19 climatic variables in Southeast Asia. [file 12711_2025_989_MOESM10_ESM.tif]
